# Elements of Pathological Anatomy, Illustrated by Numerous Engravings

**Published:** 1840-09

**Authors:** 


### REVIEWS.

---

Art. VI.—*Elements of Pathological Anatomy, illustrated by numerous engravings.* "In Morbis, sive acutis, sive chronicis, viget occultum, per humanas speculationes fere incomprehensible."—Baglivi. By Samuel D. Gross, M.D. Late professor of General Anatomy, Physiology, and Pathological Anatomy, in the Medical Department of the Cincinnati College. Vol. II, 8vo., Boston, 1839. Marsh, Capen, Lyon & Webb and James B. Dow.

---

(*Continued from the August number.*)

---

We pass by the next three chapters on hœmorrhage, softening and gangrene, to trace out the progress of purulent secretion through ulceration, granulation and cicatrization, all of which display the presence of pus. Ulceration implies excessive absorption, in connexion with purulent secretion. This remark must, however, be understood with some limitation. During the suppurating process, before the opening of an abscess, we have no evidence of increased absorption, but the reverse; and, subsequently, while the cavity is filling up, absorption does not appear to be active; again, parts which have lost their vital properties slough off, leaving excavations or depressions, that have not depended on absorption: in many cases, moreover, absorption is deficient and the granulations rise above the proper level. Some, particularly the last of these suppurating surfaces are ulcers. Our author does not believe in ulceration without inflammation conjoined with increased absorption, but concedes that the secretion of pus is not necessary.

We do not, however, believe in the existence of those cases. The purulent secretion may be scanty, but analogy justifies the conclusion, that in all cases where inflammation and excited absorption, by their combined action, produce an ulcer, that is, the destruction of the surface of a tissue, there is purulent secretion. Surgery is often called to grapple with ulcerative action, and sometimes foiled. Ulcers occasionally spread far and wide, in despite of every effort. In many of these cases, the constitution is in fault; in others the ulcerative action is specific.

Granulation, the subject of the next chapter is, intimately associated with this. Granulation exhibits two secretions, the lymphatic and the purulent. Coagulable lymph is the material out of which the granulations are formed, and into which the vessels that supply them so liberally with blood, extend from the surrounding parts; but these granulations are themselves secreting surfaces, and the fluid *they* pour out is pus, perhaps also lymph. The surgeon judges of their healthy condition, as he judges of the state of other secreting organs—by the quality of the discharge.

The natural and desirable termination of this series of sanito-morbid actions is in cicatrization or skinning, which makes the subject of the next chapter; and from which, for the purpose of varying the style of our analysis, we shall make a short extract.

"Cicatrization is the process which nature employs to heal wounds and ulcers. It is the finishing stroke, if the expression be allowable, of granulation,—the labor which is necessary to polish the surface of the sore, to contract its diameter, and to bring it as nearly as possible to a level with the surrounding structures. This process, although it is not limited to the skin, as might be inferred from reading some modern treatises on surgery, is yet most advantageously studied there, as it enables us to follow nature as it were in the different steps which she employs with a view of accomplishing her enterprise.

"The first step in the healing of an ulcer seems to be the subsidence of the inflammation, which becomes gradually less and less, until the surrounding parts regain their natural color, form, and consistence. The sore at the same time sensibly diminishes in diameter, by the contraction and coalescence of its granulations; and its surface, instead of being rough and uneven, assumes a smooth, glassy appearance, its centre, however, being still considerably depressed; or, if the granulations have been very exuberant, unnaturally elevated. Cicatrization is now observed to begin, the first indication of it being a thin, delicate, bluish pellicle, placed along the margin of the breach, where it soon unites with the old skin by an interchange of vessels, nerves, and absorbents. If the part be inspected at a later period, the substance that was thus deposited and organized, will be found to have increased in thickness and density, and to be gradually extending itself towards the centre of the ulcer by the addition of new matter. It is in this manner, by this successive experipheral action, that the denuded surface is eventually covered over."

If we are not greatly mistaken, cicatrization sometimes commences on the disks of ulcers, at a distance from their margins.

We cannot dwell longer on these chapters, all of which abound in elementary truths of great moment to the surgeon; and shall pass to some other modes of termination, or effects of inflammation.

Inflammations of intense violence are liable to end in gangrene. It is common to say, that if the constitution is feeble, a slighted inflammation may destroy the irritability and organization of the part; but these inflammations, in their intensity, bear perhaps the same relation to the powers of the system, as the former. It is not uncommon for medical men to speak of *a* gangrenous inflammation, but this is generally a misnomer. It is correct to speak of *a* gangrenous *termination* of inflammation.

"When this event is about to take place, the affected structure loses its sensibility, it becomes cold, the blood ceases to circulate, and absorption is suspended.

"The process by which these changes are accomplished is generally progressive, its rapidity varying with the constitution of the patient, the violence of the exciting causes, and, above all, the nature of the suffering structure. Thus, gangrene, in some cases, takes place in the course of a few hours, whilst, in others, it does not make its appearance for several weeks or even months from the commencement of the inflammation. Much diversity prevails amongst the different organs and tissues in regard to their liability to become affected with this lesion. The cellular, cutaneous, and mucous, may be enumerated as the textures which are more frequently seized with mortification than any other; and it is worthy of remark that these are parts which are extremely well supplied with blood, especially the two latter. Nevertheless, in the skin and cellular substance, this event takes place most frequently in situations which are remote from the central organ of the circulation, as on the hands, feet and posterior portions of the trunk. In the mucous system, the parts most liable to mortification are the gums, the inside of the cheeks, the tonsils, the colon, the inferior third of the ileum, the urinary bladder, and the lining membrane of the vulva. The serous membranes, muscles, ligaments, tendons, aponeuroses, and cartilages are rarely affected in this way; and the same remark holds good in reference to the arteries, veins, and absorbents. The three latter of these structures, indeed, seem to possess a most astonishing conservative power, and hence it is not uncommon to find them retain their integrity in the midst of the sphacelated part. In malignant scarlet fever, attended with mortification of the tonsils and upper part of the neck, I have several times seen the common carotid go on in the performance of its function, and the individual recover, notwithstanding the detachment of immense sloughs of the skin and cellular substance; and similar phenomena have often been witnessed in gangrene of the inferior extremities."

When a part mortifies, the vital activity is greater as we advance in all directions from the dead centre. The arrest of the surrounding inflammation, is the arrest of the gangrene. An ulcerative action is then set up, and the decomposed parts are cut loose from the sound. No hœmorrhage follows, because the larger arteries leading to the part have been converted into impervious cords, by the stasis of the blood in their extremities and the adhesion of its fibrin to their internal parietes.

Gangrene does not always depend on violent inflammation from common causes, but occasionally on specific influences acting on the constitution. This is what happens when ergot is used with bread. In these cases, the parts exhibit a certain degree of pain, heat and redness, before they become gangrenous. The *gangrena senilis*, sometimes scarcely manifests the slightest degree of precursory inflammation, as we had an opportunity not long since of observing in a fatal case of that malady, commencing in the heels of an elderly woman and extending forward and upward. We had not the privilege of examining the parts after death. This variety of gangrene has been said to arise from obliteration of the arteries, but is it possible, when obliteration exists, that it is an effect rather than a cause, seeing that the same vessels become obliterated in ordinary mortification?

Another effect of inflammation is *softening*, to which our author has devoted a short chapter. He regards it as among the most unequivocal signs of previous inflammation—that is, one of the legitimate effects of that mode of morbid action, especially when acute. We may assume, that inflammation which occasions softening, does not cause much lymphization. In softening the tissue tends in its organization to that of some other resembling it, but naturally less firm.

Thus bone becomes cartilaginous; cartilage is made to resemble fibro-cartilage; cartilage, fibrous membrane ; and this, as well as the serous and mucous membranes, to assume some of the qualities of the cellular. It is obvious, that deposites of coagulable lymph would retard, rather than promote this series of changes. Softening is sometimes the only test of pre-existent inflammation in the brain and gastro-enteric mucous membrane, and hence it is of great importance for the pathological anatomist to have an accurate knowledge of the variations of normal density exhibited by those tissues.

Softening, as we have already said, does not always depend on inflammation. Whatever impairs the nutrition of a tissue, will tend to produce its decomposition.

Induration is in some degree the opposite of mollescence, *ramollisement* or softening, and often shows itself as governed by the same law. Thus cellular may be converted into fibrous membrane, cartilage into bone, &c. But in other cases the tissues are consolidated with each other by deposits among them of plastic lymph. In speaking of the causes of this pathological state Prof. Gross remarks:

"These are referable, for the most part, to inflammation, followed by an effusion of coagulating lymph into the instertitial substance of the affected organ. In the lungs there is frequently, in addition to this, more or less blood poured out, which, combining with the natural structures, gives them a red color. It is thus that red hepatization is established. In chronic cases, on the other hand, the induration is commonly effected by the lymph alone; and hence it is that the organ is usually of a much lighter hue. In the hardening of the subcutaneous cellular tissue of infants, a disease of pretty frequent occurrence in certain districts of Europe, the effused matter is generally impregnated with two coloring principles, the one of an orange red, the other of a bluish shade, both of which are stated by M. Chevreul to exist in the blood."

The chapter on hæmorrhage is full of interest, but we have not space for its analysis. Professor Gross is of opinion, that in most cases not dependent on a mechanical lesion of the vessels, the hœmorrhagic action is more or less inflammatory. In evidence, among other facts, he cites the constitutional and local symptoms attendant on menstruation, especially when painful. We are aware, that many cases of the latter are inflammatory, but in these the discharge is generally sparing, whereas it ought to be copious, if caused by an inflammatory action. It is impossible to believe, moreover, that inflammation attends the profuse hœmorrhages connected with the final cessation of the menses, inasmuch as none of the ordinary symptoms of inflammation, either pelvic or constitutional, are present; and the discharge is best restrained by a treatment, the reverse of that which is adapted to the cure of inflammation. Hœmorrhages from the other mucous membranes are often mere exhalations; but in hæmoptysis the discharge is not always from the bronchial membrane. We recollect a case in which it came from an artery of considerable size, divided by ulceration, and remaining pervious. We are quite convinced that many hœmorrhages depend on a morbid state of the blood—perhaps on a diminution of its albuminous ingredients, whereby it passes through tissues, which were only competent to its confinement while it remained in a normal state.

Hypertrophy and atrophy are the subjects of separate chapters. We can but glance at them. Hypertrophy may be either general or topical. The last by far the most interesting to the physician and surgeon, generally arises from too great a determination of blood to the part. This may be the result of an irritation maintained within it for some time, raising more or less of inflammatory action; or of its being thrown into excessive action. The heart may furnish an example of both. Under the influence of certain passions, that organ becomes hypertrophied; and again, when the valves, seated at the outlet of any of its great cavities, become impaired in their structure, the parietes, from the increased labor they are called upon to perform, acquire abnormal dimensions. We lately saw a heart in the possession of Dr. Enders, in which this was beautifully illustrated by the valvular vegetations, which obstructed the passage of the blood through the organ.

Atrophy, the opposite of hypertrophy, often coexists with it in the same compound organ. Thus while one tissue is reduced below its proper size, another may be increased beyond—and *vice-versa.* More commonly, however, all the tissues of an organ are equally reduced. Atrophy implies deficient nutrition, or excessive absorption. Pressure external or internal frequently leads to it, and is said, in the common parlance of the profession, to do so by promoting absorption. In these cases, however, the supply of blood is limited by the compression of the nutrient arteries. In this manner portions of the lung and liver are occasionally greatly reduced in size—contracting coagulable lymph having been effused about and within them. The causes of atrophy are, however, extremely various. Thus the disuse or rather non-use of an organ or a limb rapidly reduces its size; and a lesion of its innervation, impairing its function of nutrition, may have the same effect. In old age nearly all the parts of the body pass into a state of atrophy; on the other hand, the whole never rise into hypertrophy. The great bulk which some aged persons exhibit, arises from hypertrophy of the adipose tissue alone.

Our author next arrives at transformations—the conversion of one texture into another. He regards the whole as

"effected under the influence of inflammatory irritation." To this sweeping generalization we are not quite prepared to give our assent. Nevertheless, many transformations take place almost under our eyes, and can be seen to depend on inflammatory action. Thus when the skin is transformed into mucous membranes, it is under circumstances of irritation, and with the appearance of inflammation; when a deep seated abscess does not fill up with granulation and a fistula remains, this artificial efferent duct comes to be lined with a mucous membrane, analagous to that of the excretory vessels of certain glands,—which may be regarded as the transformation of cellular into mucous membrane. A few months since we saw an extensive adipous transformation of the gastrocnemii and other muscles of the leg, in connexion with chronic inflammation and articular ulceration of the ankle, rendering amputation necessary. The skin of the same limb was changed into a tissue nearly resembling cartilage.

Many transformations, however, are not preceded by symptoms of inflammation. Such are ossifications of the cartilages of the ribs, the valves of the heart, and the parietes of the vessels—on the other hand, bones occasionally pass into the state of cartilage, without the signs of inflammation being present. Transformation is, in fact, a lesion of nutrition and may take place with or without inflammation.

The following are the analagous transformations admitted by our author: 1. the cellular—2. the mucous—3. the cutaneous—4. the fibrous—5. the cartilaginous—6. the osseous—7. the adipous. These cannot be formed in or out of any other tissue of the body, indiscriminately. The law which governs their production, is nearly the same that presides over the softening of the tissues. The cellular may pass into the mucous, and this into the dermoid—or into the fibrous which may then become fibro-cartilaginous, cartilaginous and osseous.

Under no other aspect does the science of general anatomy appear more attractive and useful, than when administering to the study of these transformations; the contemplation of which fills the mind with cheering anticipations of the future condition of pathological science. D.

